# The Novel Anti-cMet Antibody seeMet 12 Potentiates Sorafenib Therapy and Radiotherapy in a Colorectal Cancer Model

**DOI:** 10.3389/fonc.2020.01717

**Published:** 2020-09-11

**Authors:** Diana Spiegelberg, Anja Charlotte Lundgren Mortensen, Kartika Dyah Palupi, Patrick Micke, Julin Wong, Borivoj Vojtesek, David Philip Lane, Marika Nestor

**Affiliations:** ^1^Department of Immunology, Genetics and Pathology, Uppsala University, Uppsala, Sweden; ^2^Department of Surgical Sciences, Uppsala University, Uppsala, Sweden; ^3^p53 Laboratory, Agency for Science, Technology and Research (A^∗^STAR), Singapore, Singapore; ^4^Research Centre for Applied Molecular Oncology (RECAMO), Masaryk Memorial Cancer Institute, Brno, Czechia; ^5^Department of Microbiology, Tumor and Cell Biology, Science for Life Laboratory, Karolinska Institutet, Stockholm, Sweden

**Keywords:** radio-sensitization, chemo-sensitization, combination treatment, colorectal cancer, c-Met, HGFR, synergy

## Abstract

**Rational:**

cMet is abnormally regulated in gastrointestinal cancer, and is associated with increased invasiveness of the disease and poor overall survival. There are indications that targeted therapy against cMet, alone or in combination with additional cancer therapies, can help improve treatment outcome. Thus, in the present study we investigated the therapeutic efficacy of a novel cMet-targeting antibody therapy in gastrointestinal cancer models, and assessed potential augmenting effects in combination with tyrosine kinase inhibitor (TKI) targeted therapy or radiotherapy.

**Methods:**

Three different cMet-targeting antibodies were first characterized with respect to antigen binding and effects on cell viability *in vitro*. The best performing candidate seeMet 12 was then further assessed for effects on colorectal cancer cell growth, proliferation and migration. Combinations with the TKI-inhibitor sorafenib or external beam radiotherapy were then evaluated for potential additive or synergistic effects *in vitro* using monolayer- and multicellular tumor spheroid assays. Finally, the combination of seeMet 12 and radiotherapy was evaluated *in vivo* in a proof-of-concept colorectal cancer xenograft study.

**Results:**

Dose-dependent therapeutic effects were demonstrated for all three cMet-targeting antibodies. Monotherapy using seeMet 12 resulted in impaired cellular migration/proliferation and reduced tumor spheroid growth. Moreover, seeMet 12 was able to potentiate therapeutic effects *in vitro* for both sorafenib and radiotherapy treatments. Finally, the *in vivo* therapy study demonstrated promising results, where a combination of seeMet 12 and fractionated radiotherapy increased median survival by 79% compared to radiotherapy alone, and tripled maximum survival.

**Conclusion:**

The novel anti-cMet antibody seeMet 12 demonstrated therapeutic effects in cMet positive gastrointestinal cancer cells *in vitro*. Moreover, the addition of seeMet 12 augmented the effects of sorafenib and radiotherapy. An *in vivo* proof-of-concept study of seeMet 12 and radiotherapy further validated the results. Thus, cMet-targeted therapy should be further explored as a promising approach to increase therapeutic effects, circumvent treatment resistance, and reduce side effects.

## Introduction

The tyrosine-protein kinase Met (cMet), also known as hepatocyte growth factor receptor (HGFR), is a heterodimer transmembrane tyrosine kinase receptor encoded by the *MET* proto-oncogene. The natural ligand for the cMet receptor is hepatocyte growth factor (HGF), an inactive protein which is changed into its active form by proteolytic cleavage. After binding to HGF, cMet dimerizes and triggers transphosphorylation in the catalytic domain, eventually opening the cMet active docking sites^3^. cMet activation activates multiple signal transduction pathways involved in regulating motility, proliferation and survival, such as RAS, PI3K, STAT, beta-catenin and Notch pathways (1).

Abnormal regulation of cMet has been reported in several types of cancers, including colorectal cancer, non-small cell lung cancer, gastric carcinoma and breast cancer (2–5). High activation of cMet and its downstream signaling pathways has been demonstrated to trigger hyperproliferation, tumor invasion, angiogenesis, and correlates with poor survival (6). Various processes such as engagement with additional cell surface receptors, elevated ligand stimulation, mutations, and overexpression of the cMet receptor may stimulate this aberrant signaling of cMet (1).

In addition to its role as an oncogenic driver, increasing evidence implicates cMet as a central factor in resistance to chemotherapy and radiotherapy, as well as to targeted therapies toward e.g., VEGFR and EGFR. Suggested mechanisms include promotion of an invasive growth program, and/or induction of stem cell-like properties, and mediating protection from apoptosis (7–10). Consequently, it is not surprising that inhibition of the cMet signaling pathway is being increasingly investigated as a mechanism to target for the development of new anticancer agents. This may be a way to potentiate existing targeted therapies, as well as for preventing or reversing drug resistance. Sorafenib resistance is one example where recent studies have implicated cMet activity as a main resistance factor with important clinical implications (11, 12). Sorafenib is a recently introduced small-molecule multi kinase inhibitor, currently approved for treatment of e.g., advanced renal cell carcinoma and hepatocellular carcinoma (HCC), and is currently in clinical trials for treatment of e.g., colorectal cancer (13). It inhibits multiple kinases involved in tumorigenesis (Raf-1, wild type B-Raf, mutant B-Raf, c-Kit, Flt-3, and RET) (14), as well as proangiogenic receptor tyrosine kinases including VEGFR-1/2/3, PDGFR-β, and FGFR1. However, low and unstable response rates and short effective duration in clinical trials (15) suggest intrinsic primary and acquired secondary resistance. New therapeutic approaches or rational drug combinations are consequently important to explore for improving the clinical benefits of this drug (16), and cMet inhibition may be a very interesting strategy due to the aforementioned potential role of cMet in sorafenib resistance.

Furthermore, cMet inhibition has also been suggested as a potential route for augmenting radiotherapy and mitigating radiation resistance. Radiation, alone or in combination with chemotherapy, remains the foundation of treatment for various solid tumors, including breast, lung, urological and lower gastro-intestinal cancers (17). However, due to the proximity of critical normal tissues and tumor radioresistance, curative radiation doses are not always reached. Irradiation has been shown to induce overexpression and activity of cMet by the induction of ATM kinases and NF-κB, to protect cells from DNA damaging agents. Studies suggest that cMet participates in radiation-induced progression through the epithelial-mesenchymal transition (EMT), mediating radiation resistance (10). Moreover, cMet activation, via PI3K and AKT signaling pathways, has been shown to protect cells from radiation-induced apoptosis (8, 10, 18–20). In a recent study, the HGF/cMet signaling pathway was found to be activated in schwannomas resistant to radiotherapy, which could be overcome by cMet blockade (21). These studies suggest that combined treatment with cMet inhibitors may enhance radiosensitivity and circumvent the onset of radiation resistance.

Due to its involvement in oncogenic pathways and drug resistance, the Met-HGF axis has been under exploration as a cancer drug target. Besides various MET kinase inhibitors, there have been several reports of cMet inhibition by anti-Met antibodies, primarily by interfering with the HGF:MET complex with various success. The monovalent 5D5 antibody (MetMab), presently in clinical trials (22), competes with HGF for cMet binding, while the monovalent DN-30 antibody fragment inhibits cMet signaling by cMet receptor down regulation (23). Bivalent LMH 87 antibody was shown to cause cMet down regulation by receptor internalization (24). Given the recent successes of therapeutic antibodies, as well as the accumulating evidence of cMet involvement in cancer development, the outlook for developing anti-cMet therapeutic antibodies is promising.

We have recently developed a panel of non-agonistic anti-cMet monoclonal antibodies, referred to as Specifically Engaging Extracellular cMet antibodies (seeMet) (7). The antibodies were shown to bind with high affinity and specificity to the α-chain of cMet. In the present study we have assessed three of these novel cMet-targeting antibodies (seeMet 12, seeMet 13, and seeMet 18), binding to three different regions of cMet, for their therapeutic potential in gastrointestinal cancer models. Moreover, the best performing candidate seeMet 12 was then further characterized for potential combination effects with either the TKI sorafenib or with external radiotherapy in both monolayer- and multicellular tumor spheroid colorectal cancer models. Finally, the combination of seeMet 12 and radiotherapy was evaluated in a proof-of-concept therapeutic study in colorectal cancer xenografts.

## Materials and Methods

### Cell Culture and Maintenance

HT-29, a cMet positive human colorectal adenocarcinoma cell line with KRAS wild-type and BRAF^V600E^ mutation (obtained from the American Tissue Culture Collection (ATCC), were cultured in McCoy’s 5A modified medium (Biochrom GmbH, Germany). Media was supplemented with 10% (v/v) fetal bovine serum (Sigma Aldrich, United States), L-glutamine and antibiotics (100 IU penicillin and 100 μg/ml streptomycin) from Biochrom GmbH (Berlin, Germany). The gastric adenocarcinoma cell line MKN-45, obtained from Deutsche Sammlung von Mikroorganismen und Zellkulturen GmbH (DSMZ, Braunschweig, Germany) was cultured in RPMI 1640 medium, supplemented with 20% FBS, L-glutamine and antibiotics. All cell lines distributed by the ATCC and DSMZ are tested mycoplasma negative by at least two different assays before distribution. Monolayer stock cultures were grown in 25 or 75 cm^2^ tissue culture flasks (VWR, United States). Passaging was performed using trypsin-EDTA (ethylenediamine tetraacetate) (Biochrom GmbH, Germany) after the cell culture reaching 80–90% confluency. Cells were incubated at 37°C in an atmosphere containing 5% CO_2_.

### Antibodies

Selection of a panel of 11 antibodies to cMet have been previously described (7). Three of the hybridoma clones seeMet 12, seeMet 13 and seeMet 18 adapted well to growth in serum free medium allowing exceptionally clean purification of the antibodies. The mouse monoclonal anti-cMet antibodies seeMet 12, seeMet 13, and seeMet 18, were produced at the Research Centre For Applied Molecular Oncology (RECAMO), Masaryk Memorial Cancer Institute, Czech Republic. SeeMet 12, seeMet 13 and seeMet 18 were grown in HYBRIDOMA-FCS (Gibco, cat. no. 12 300-067) and purified using high salt (25) method and protein A-column (Sigma). Buffer exchange was performed using Zeba Spin Desalting Columns 7 K MWCO (Thermo Scientific cat. no. 89892). Molarity calculations were performed according to: molarity = concentration/molar mass (150,000 Da).

Characterizations of the panel of 11 antibodies to cMet have been previously described (7). In brief, the earlier characterizations of seeMet 12 demonstrated outstanding cMet specificity toward purified cMet α-chain in Western blot analysis, where transfected as well as endogenous human cMet was successfully detected with a higher binding affinity observed than for seeMet 13 and seeMet 18. SeeMet 12 also successfully detected mature cMet α-chain and precursor cMet in immunoprecipitated cell lysates, whereas binding to native cMet in flow cytometry of SNU-5 was weak. The sequence of binding region was determined by pepscan analysis to LEHPDCFPCQDCSSK. Characterization of seeMet 13 demonstrated the strongest binding to native cMet in flow cytometry of SNU-5 cells, whereas specificity and affinity toward cMet was poor on Western blots. Cell studies on seeMet 13-treated cells demonstrated reduced cell division, decreased binding at lower temperatures, and indicated internalization upon binding. The sequence of binding region was determined to FRDS. Characterizations of seeMet 18 successfully detected purified cMet α-chain, and transfected as well as endogenous human cMet in Western blot experiments, with a higher binding affinity observed than for seeMet 13. Some cross reactivity to cMet null cells was however, evident. seeMet 18 also detected mature cMet α-chain and precursor cMet in immunoprecipitated cell lysates, whereas binding to native cMet in flow cytometry was weak. Cell studies on seeMet 18-treated cells demonstrated no impact on cell division, and indicated decreased binding at lower temperatures. The sequence of binding region was determined to LVVDTYYDDQ.

### XTT Cell Proliferation Assay

The cell viability of HT-29 and MKN-45 cells was assessed using sodium 2,3-bis(2-methoxy-4-nitro-5-sulfophenyl)-5-[(phenylamino)-carbonyl]-2*H*-tetrazolium (XTT) cell proliferation assay kit (ATCC^®^, United States). The cells were seeded in 96-well plates, in concentrations of 5,000 and 2,000 cells per well for HT-29 and MKN-45 cells respectively, at 37°C in an atmosphere containing humidified air with 5% CO_2_, 48 h before treatment of >3 wells with 0–250 nM of seeMet 12, seeMet 13, and seeMet 18 (HT-29 nd MKN-45) or 0–10 μg/ml sorafenib (HT-29) in cell culture medium.

The XTT assay was performed according to manufacturer’s instruction. Briefly, 5 ml XTT reagent and 100 μl of *N*-methyl dibenzopyrazine methyl sulfate activation reagent was added to every 10 ml medium needed. After treatment, the medium was discarded from the wells and replaced with 150 μl XTT-solution/well). The absorbance was measured after 4 h incubation times using a microtiter plate reader (BioRad, United States) at 450 nm (specific absorbance) and 655 nm (non-specific absorbance). The specific absorbance was calculated as follows: Specific Absorbance = A450 nm (Test) –[A450 nm (Blank)-A655 nm (Blank)] – A655 nm (Test). The percentage of inhibition was calculated as follows: (Mean absorbance of treated cells)/(Mean absorbance of control cells) × 100%.

### Radiolabeling

The antibodies seeMet 12, seeMet 13 and seeMet 18 (1 μg/μl, in borate buffer, pH 9) were incubated with CHX-A”-DTPA (1 mg/ml in borate buffer, 0.07 M, pH 9) in a molar excess of 5:1 (CHX: antibody) for 4–16 h at 37°C. The conjugated antibodies were then separated from free CHX-A”-DTPA using a NAP-5 column equilibrated 0.2 M ammonium acetate, pH 5.5, stored over Chelex 100. Between 1 and 20 MBq ^177^Lutetium Chloride (177Lu Perkin Elmer, Sweden) was mixed with 10–120 μg chelated antibody and incubated for 60 min in 37°C. 0.5 μl ^177^Lu-labeled seeMet antibody was added to an ITLC-strip, with 0.2 M citric acid or sodium citrate as mobile phase, and was analyzed in a phosphorimager. If necessary, labeled antibodies were separated from non-reacted ^177^Lu and low-molecular-weight reaction components by using a NAP-5 column pre-equilibrated with PBS. All experiments were performed with purity of >90%.

### Radioimmunoassay

HT-29 and MKN-45 cells were harvested by trypsinization and seeded in concentrations of 50,000–75,000 cells per well in 48-well plates. After 24 h incubation (at 37°C, 5% CO_2_), 30 nM ^177^Lu-labeled antibody or 30 nM ^177^Lu-labeled antibody together with >20-fold blocking solution of unlabeled antibody was added to >3 wells each. After 24 h, the radioactive media was removed and cells were washed 2–3 times with supplement free cell media, followed by trypsinization and cell counting. The cells containing media was measured in a gamma counter (1480 Wizard 3”, Wallace, Turku, Finland).

### Western Blot

HT-29 cells were grown in monolayer and incubated with 0, 100, 200, and 400 nM seeMet 12. After drug incubation of 72 h, cell lysates were prepared according to standard protocols. The samples were separated on a NuPAGE 4-12% Bis-Tris gel (Novex, United States) using 1× MES SDS running buffer (Novex, United States) and transferred by wet blotting to a membrane (Immobilon, Millipore, Bedford, MA, United States) using 20% methanol transfer buffer for 2 h at 4°C. The membrane was blocked using 5% BSA in PBS with 1% Tween 20 for 1 h and then incubated with primary antibodies on a shaking plate at 4°C overnight. The primary antibodies used were Anti-Met (ab51067,abcam, United States, 1:1000), Anti-pMet (#3077S, Cell Signaling, 1:200), Anti-AKT (ab179463, abcam, United States,1:10,000), Anti-pAKT (Thr 308) (#9275S, Cell Signaling, United States, 1:700), Anti-ERK (ab54230, Abcam, United States, 1:2,000), Anti-pERK (ab214362, Abcam, United States, 1:500), Anti-BAD (D24A9, Cell Signaling, United States, 1: 700), Anti-pSTAT3 (EP2147Y, ab76315, Abcam, United States, 1:100,000), Anti-mTor (#2983, Cell signaling, United States, 1:1000), Anti- beta Actin (Sigma Aldrich, Germany, 1:100,000) and Anti-Sodium Potassium ATPase Antibody (EP1845Y, ab76020, abcam, United States, 1:100,000) - Plasma membrane charge control (1: 5000) (Abcam, United Kingdom). After the membrane was washed three times with PBS-T, the membrane was incubated with secondary antibodies. HRP goat anti-mouse (1: 10,000) (Invitrogen, United States) and HRP goat anti-rabbit (1: 30,000) (Invitrogen, United States) were used as secondary antibodies. The band visualization was performed by incubating the membrane in an electrochemical luminescent solution (Immobilon, Millipore, Bedford, MA, United States) for 60 s, and pictures of the bands were recorded using a CCD camera (SuperCCD HR, Fujifilm, Japan).

### Flow Cytometry

HT-29 cells were treated with 0, 100 and 200 nM of seeMet 12. 96 h post treatment cells were harvested with trypsin-EDTA (Biochrom GmBh, Gemany), rinsed with PBS and stained with the CellEventsTM caspase-3/7 green flow cytometry assay kit (Thermo Fisher, Sweden) according to manufacturer’s instructions. Samples were analyzed on a CyFlow^®^Space flow cytometer (Sysmex, Japan) and at least 10,000 events per sample were collected. Data analysis was performed using FlowJo^TM^ Software v10.6.1, (for Windows) Becton, Dickinson and Company; 2019.

### Migration/Proliferation Assay

The cell migration and proliferation ability of HT-29 cells was studied using a wound healing assay (also called scratch assay), as previously reported (26). In short, cells were grown at confluence in 6 well plates and a narrow area on the monolayer was scratched off with a p10 pipette tip. Afterward, wells were washed and incubated with normal cell medium, 100 or 250 nM seeMet 12. Images from the same scratch location were obtained directly after scratching, 24, 48, and 72 h of incubation using an inverted microscope Nikon Diaphot (Nikon, Japan) mounted with Canon EOS 700D camera (Canon Inc., Japan). Migration distance was measured and analyzed using ImageJ 1.51k software (NIH, Bethesda, MD, United States). The experiments were repeated 4 times.

### Clonogenic Assay

HT-29 cells were seeded in T25 or 6-well plates (VWR, United States) in multiple cell concentrations, increasing with drug and radiation dose (0 Gy: 250–750, 2 Gy: 1,000–2,500, 4 Gy: 2,500–5,000, 6 Gy: 5,000–10,000, 8 Gy: 10,000–12,000, Sorafenib: 500–1,800 cells/well). After 24 h, cells were treated with 0, 100, or 250 nM seeMet 12 alone or in combination with other treatments (2 μg/ml sorafenib, 5 μg/ml sorafenib or radiation (0, 2, 4, 6, 8 Gy) and incubated at 37°C in an atmosphere containing 5% CO_2_. After about 15 days, the medium was discarded, cells were washed with PBS and fixed using 95% ethanol for 10–20 min. Colonies were stained with hematoxylin (Histolab, Sweden) for 20 min, put in the water bath with tap water for 30 min, and let dry overnight. Colonies (defined as cell clusters that consist of >50 cells) counted. Plating efficiency (PE) and survival fraction (SF) were calculated as follows: PE = (number of colonies formed)/(number of cells seeded) × 100% and SF = (number of colonies formed after treatment)/(number of cells seeded × PE). Irradiation of the seeded cells was performed using a X-Rad 225 IR irradiator (Precision X-Ray Inc., Germany) with a 0.3 mm Cu filter, rotating table and a dose rate of 1 Gy/min. Experiments were repeated 4 times.

### 3D Tumor Spheroids

3000–4000 HT-29 cells/well were seeded in 96-well flat bottom plates (Sarstedt, Germany) coated with 50 μl 0.15% agarose (Sigma Aldrich, United States) and incubated for 72 h. Established spheroids [mean spheroid starting sizes of 0.030 mm^3^ ± 0.005 (SD) mm^3^] were treated with (a) 100, 250 or 400 nM seeMet 12 monotherapy, (b) 2 and 5 μg/ml sorafenib monotherapy and the combination of sorafenib and 100 and 250 nM seeMet 12 or (c) 2, 4, and 6 Gy radiation and the combination with 100 and 250 nM seeMet 12. Twice a week, half of the incubation medium was replaced with fresh medium. Spheroids were followed for >14 days and pictures were taken 3–4 times a week using an inverted microscope Nikon Diaphot (Nikon, Japan) mounted with Canon EOS 700D camera (Canon Inc., Japan). Spheroid sizes were measured and analyzed using ImageJ 1.51k software (NIH, Bethesda, MD, United States). Irradiation of the multicellular tumor spheroids was performed using X-ray irradiation with a Linear accelerator “Elekta Precise Treatment System” at the unit for radiotherapy treatment (Strålbehandlingsavdelningen) at Uppsala University Hospital. The dose rate was 5 Gy/min.

### *In vivo* Xenograft Study

The *in vivo* study was conducted in accordance with the recommendations of FELASA and complied with Swedish law, and with approval of the Uppsala Committee of Animal Research Ethics. Female nu/nu Balb/c mice (*n* = 16, age = 4–6 weeks) were maintained under standard laboratory conditions and fed *ad libitum*. The mice were injected subcutaneously into the right flank with 5 × 10^6^ HT-29 cells. After tumors established, the tumor growth and body weight were monitored every day. The tumor volume was calculated with V = 4πa × b × c/24 where a, b, and c are the diameter in all dimensions. One mouse was excluded from the study due to lack of tumor take. Eleven days after tumor implantation, animals were randomized to receive one of the following treatments: (1) control (*n* = 4); (2) radiotherapy 2 Gy on three consecutive days (*n* = 3), (3) seeMet 12 therapy 100 μg (i.v.) on five consecutive days (*n* = 4) and (4) combination therapy 5× seeMet 12 and 3 × 2 Gy. (*n* = 4). Mice in the control group and in the radiotherapy group received saline injections (placebo), according to the drug schedule. The radiotherapy fractions were given 24, 48, and 72 h after the first seeMet 12 or placebo drug dose. Mice in the radiotherapy and in the seeMet 12 and radiotherapy group were anesthetized with isoflurane and imaged with a cone beam computed tomography (CBCT) scan prior radiotherapy using the Small Animal Radiation Research Platform (SARRP) (Xstrahl Medical & Life Sciences, Germany). With the help of the CBCT scan the isocenter of the tumor was identified and aligned with the central axis of the beam. Mice were irradiated with X-ray beams at a dose of 2 Gy using the variable collimator (0.8 cm^2^ window). The mice were followed until the study endpoint of tumor size of 1,000 mm^3^ was reached.

### *Ex vivo* Immunohistochemistry

HT-29 tumors were dissected at a size of about 1,000 mm^3^ and directly transferred to formalin. After fixation the tumors were embedded in paraffin and cut in 4 μm-sections. The sections were stained with hematoxylin and eosin (Biocare Medical) using a Tissue-Tek Prisma Plus, Sakura instrument according to the manufacturer’s instructions. Immunostaining with Anti-Ki67 (DAKO, M7240) and Anti-Annexin V (abcam, ab108321, rabbit) was performed after pretreatment with Targen Retireval Solution, Low pH, Dako, K800521-2; antibody dilution 1: 1000, 30 min RT and cMet (Abcam, ab51067), pretreatment – Target Retrieval Solution, High pH, Dako, K800421-2; antibody dilution 1: 100, overnight incubation then detected with Dako EnVision Flex High pH # K801021-2 kit.

Necrotic areas were quantified by estimating the fraction of necrosis in the whole tumor area in five 10× power fields. Mitotic and apoptotic cells (Annexin V positive) were scored in ten 40× power fields. cMet expression scored according to staining intensity (negative -, weak+, moderate++, or strong+++). The proliferation index was quantified by calculating the fraction of Ki67 positive cells in an 0.25 mm^2^ grid in a hot spot area. All histopathologic evaluation was done in a blinded manner by a pathologist.

### Statistical Analysis

Statistical analysis between two groups was performed using Student’s *t*-test. Statistical analysis of three or more groups was performed using analysis of variance (ANOVA) with Tukey’s *post hoc* test. All statistical data analysis was performed using Graph Pad Prism 8 (La Jolla, United States). Data were expressed as mean ± SD (if not stated otherwise) and *p* < 0.05 considered to be statistically significant (^∗^*p* < 0.05, ^∗∗^*p* < 0.01, ^∗∗∗^*p* < 0.001, ^****^p < 0.0001). Combination effects of seeMet 12 and sorafenib, and seeMet 12 and radiotherapy in clonogenic survival assays were analyzed by the Chou-Talalay-method by the software CompuSyn 3 developed by Nick Martin of MIT, Cambridge, MA, United States. The combination index (CI) and fraction affected (Fa) was calculated and operates as the percent growth inhibition (27). A CI of ≤0.8 indicates synergism, CI ≥ 1.2 antagonism. A CI > 0.8 < 1.2 indicates additive effect.

## Results

### cMet-Targeted Monotherapy Reduces Cell Viability in 2D and 3D Colorectal Cancer Models in an Antigen Specific and Dose Dependent Manner

**FIGURE 1:**
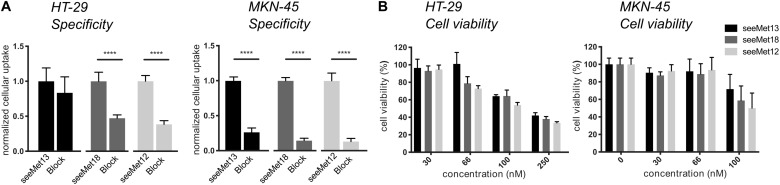
Characterizations of the antibodies seeMet 13, seeMet 18 and seeMet 12 on two gastrointestinal cancer cell lines (HT-29 and MKN-45). **(A)** Radioimmunoassays of binding specificity of radiolabeled seeMet 13, seeMet 18 seeMet 12 with and without the presence of a >20-fold molar excess of corresponding unlabeled antibody. *N* > 3. Error bars represent standard deviation (SD). **(B)** XTT viability assays. Data has been normalized to viability for untreated controls (100%). *N* > 3. Error bars represent standard deviation (SD). Considered to be statistically significant *p < 0.05, **p < 0.01, ***p < 0.001, ****p < 0.0001.

First, cMet antigen presence and antibody binding specificity was assessed on HT-29 and MKN-45 cells for all three antibodies, by comparing cellular binding of uncompeted radiolabeled antibodies to binding in the presence of >20-fold molar excess of unlabeled antibody (Figure 1A). Blocking of binding was clearly demonstrated for all three antibodies on MKN-45 cells, and on HT-29 cells for seeMet 18 and seeMet 12. The growth inhibitory effects of the three different anti-cMet antibodies were then assessed on both HT-29 and MKN-45 cells in XTT viability assays (Figure 1B). Results demonstrated dose dependent effects of all three antibodies, with seeMet 12 indicating the largest inhibitory effect.

**FIGURE 2:**
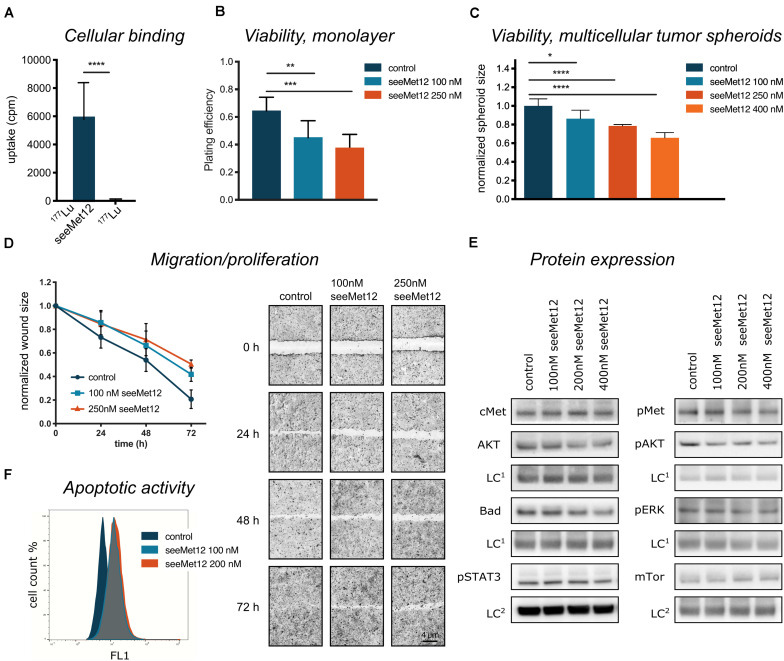
Characterization of seeMet 12 monotherapy in HT-29 cells. **(A)** Cellular binding assessed by radioimmunoassay comparing ^177^Lu-radiolabeled seeMet 12 and free radionuclide ^177^Lu. *n* = 3, Error bars represent SD. **(B)** Viability assessed in a 2D model (clonogenic survival), displayed as plating efficiency after treatment with 100 and 250 nM seeMet 12. *N* = 4, Error bars represent SD. **(C)** Viability assessed in a 3D model (tumor spheroids), displaying multicellular tumor spheroid size at day 18 after treatment with 100, 250, and 400 nM seeMet 12. *n* = 10 for control, *n* = 5 for treatment groups. Error bars represent SD. **(D)** Cellular migration/proliferation assessed with wound healing/scratch assay treated with 100 and 250 nM seeMet 12. Images of the scratch were taken at experiment start (0 h) and after 24, 48, and 72 h. *N* = 4, Error bars represent SD. Representative images from the HT-29 migration assay treated with seeMet 12, images were taken with 4× magnification. **(E)** Western blot analysis of cMet, AKT, Bad and pSTAT, pMet, pAKT, pERK and mTor 72 h after treatment with 100, 200, or 400 nM seeMet 12. The representative images were taken with the same exposure times across the entire image, however, different for different membrane fragments of the assessed proteins. The corresponding loading control (LC^1^ = Sodium Potassium ATPase; LC^2^ = beta actin) of the same membrane is displayed below the membrane of the investigated protein. **(F)** Representative histogram for flow cytometric assessment of caspase 3 and 7 (apoptotic activity) in control, 100 and 200 nM seeMet 12 treated monolayer cells. Considered to be statistically significant *p < 0.05, **p < 0.01, ***p < 0.001, ****p < 0.0001.

**FIGURE 3:**
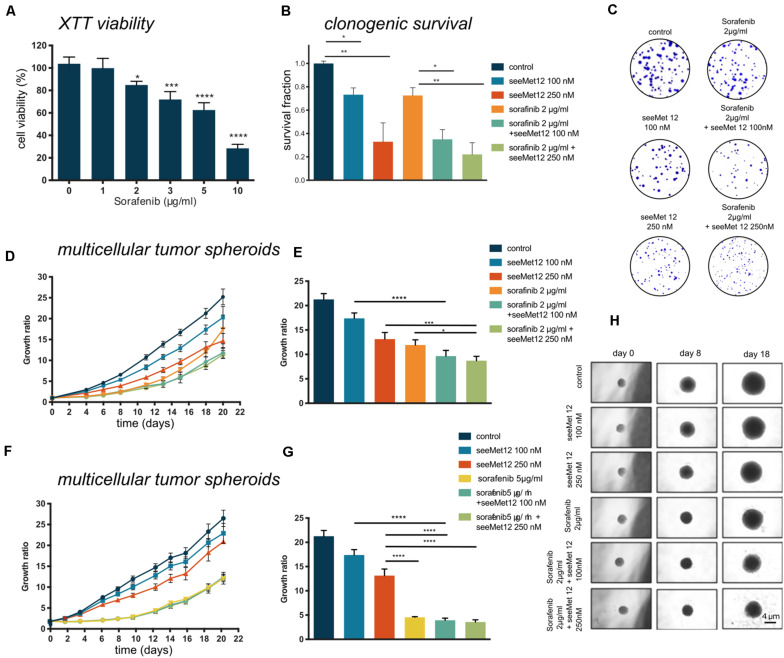
Characterization of seeMet 12 therapy in combination with sorafenib treatment. **(A)** Cell viability (XTT assay) of HT-29 cells after exposure of 1–10 μg/ml sorafenib. *N* > 3, Error bars represent SD. **(B)** Clonogenic survival assay of HT-29 cells treated with seeMet 12, sorafenib, or the combination of the two. Cells were exposed to 100 or 250 nM seeMet 12 and 2 μg/ml sorafenib, *N* = 4, error bars represent SD. **(C)** Representative images of the colonies in the clonogenic survival assay after 14 days. **(D)** Multicellular HT-29 tumor spheroid assay treated with 100 or 250 nM seeMet 12 and/or 2 μg/ml sorafenib. *n* = 10 for control, *n* = 5 for treatment groups. Error bars represent SD. **(E)** Comparison of the spheroid sizes in panel **(D)** on day, 18 Error bars represent SD. **(F)** Multicellular HT-29 tumor spheroid assay with 100 or 250 nM seeMet 12 and/or 5 μg/ml sorafenib. *n* = 10 for control, *n* = 5 for treatment groups. Error bars represent SD. **(G)** Comparison of the spheroid sizes in panel **(F)** on day 18. Error bars represent SD. **(H)** Representative images of the spheroids after the different treatments. Size reference bar = 4 μm. Considered to be statistically significant *p < 0.05, **p < 0.01, ***p < 0.001, ****p < 0.0001.

Based on this data, seeMet 12 was selected for further investigations of cellular binding and potential therapeutic effects as a monotherapy in 2D and 3D colorectal cancer models (Figure 2). A cellular binding assay (Figure 2A) further validated the antigen-specific binding for seeMet 12. Moreover, viability assays using longer time-spans validated the dose-dependent therapeutic effects seen in Figure 1B, both in 2D clonogenic survival assays (Figure 2B) and 3D multicellular tumor spheroid assays (Figure 2C), with significant reductions in viability observed at all assessed concentrations. In the 2D clonogenic assay, the plating efficiency was reduced with approximately 30 and 42% for 100 and 250 nM seeMet 12 respectively, compared to controls. In the multicellular tumor spheroid assay, spheroid sizes were reduced with approximately 14 and 21% for 100 and 250 nM seeMet 12 respectively. Moreover, wound-healing assays (Figure 2D) demonstrated that monodrug treatment with seeMet 12 decreased the migration and/or proliferation of the seeMet 12-treated cells in a dose dependent manner, further validating the effects of the antibody treatment.

### SeeMet 12 Downregulates cMet Downstream Targets and Increases Apoptosis

Molecular effects of seeMet 12 treatment was then assessed using western blot and flow cytometry (Figures 2E,F). The incubation of HT-29 cells with seeMet 12 led to slight changes in expression of the receptor kinase Met and its phosphorylated form with the greatest signal decrease at the highest concentration (Figure 2E). The expression of the downstream targets such as the cell signal proteins AKT, ERK, BAD, pSTAT3 and mTor followed this pattern with slightly lower expression with increasing doses of seeMet 12. In flow cytometry assessments, seeMet 12 treatment of HT-29 cells resulted in an increase in the expression of the apoptosis marker cleaved caspase 3/7 (Figure 2F). The mean fluorescence intensity of the marker almost doubled after exposure to 100 nM seeMet 12. A further dose increase did not increase the signal for caspase 3/7.

### SeeMet 12 Promotes Growth Inhibition of Sorafenib in 2D and 3D *in vitro* Colorectal Cancer Models

The combination effect between seeMet 12 and the tyrosine kinase inhibitor sorafenib was assessed *in vitro*. Sorafenib has previously demonstrated efficacy in both BRAF wildype and BRAF mutated cancer cell lines (14). In the present study, sorafenib reduced the HT-29 (BRAF^V600E^ mutant) cell viability in a concentration-dependent manner (Figure 3A) with IC_50_ 6.05 μg/ml after 96 h incubation time.

Growth inhibitory effects of sorafenib and seeMet 12 were then evaluated using both 2D clonogenic assays and 3D multicellular tumor spheroids. In clonogenic survival assays, the effects of 100 or 250 nM of seeMet 12 in combination with 2 μg/ml sorafenib were assessed. Significant potentiating effects with seeMet 12 were demonstrated for both seeMet 12 concentrations (Figures 3B,C), where survival fractions of sorafenib-treated cells in combination with 100 or 250 nM seeMet 12 were reduced with 65 and 78% from controls cells, and with 51 and 70% from cells treated with sorafenib alone. Synergy calculations demonstrated synergistic effects for 2 μg/ml sorafenib for both seeMet 12 concentrations, with Combination Index values of CI = 0.35 and 0.27 for 100 and 250 nM respectively.

Growth inhibitory effects in 3D multicellular tumor spheroids were then assessed for combinations of sorafenib (2 μl/ml or 5 μg/ml) and seeMet 12 (100 or 250 nM) (Figures 3D–H). Results were in line with the clonogenic survival assay, with 2 μg/ml sorafenib in combination with 250 nM seeMet 12 demonstrating the strongest potentiating effects. Sorafenib concentrations of 5 μg/ml demonstrated too strong therapeutic effects in order to discern any potentiating effects from seeMet 12.

### SeeMet 12 Potentiates Radiotherapy in 2D and 3D *in vitro* Colorectal Cancer Models

**FIGURE 4:**
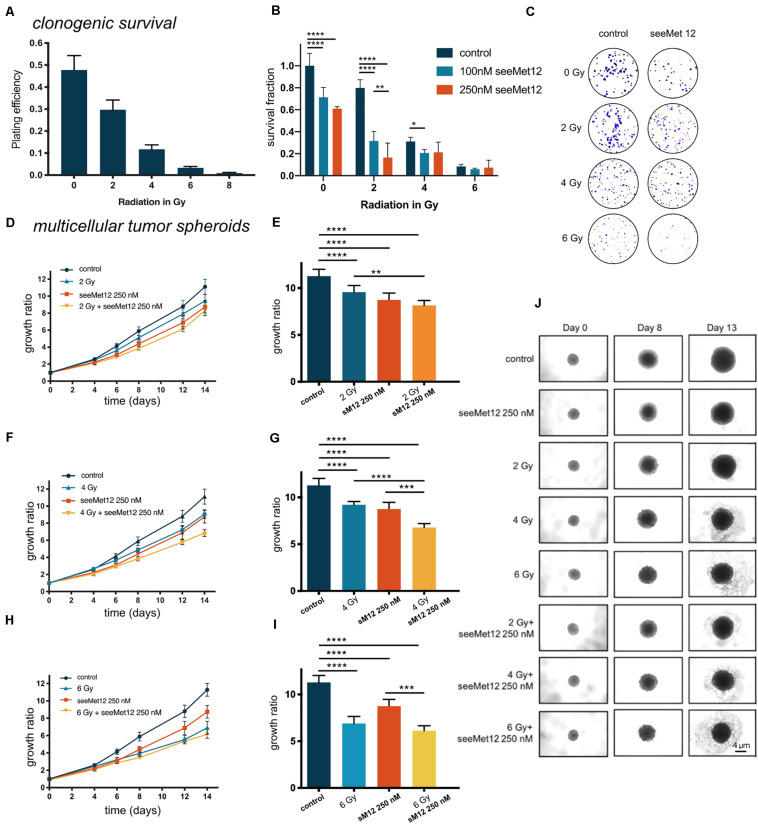
Characterization of seeMet 12 therapy in combination with external radiotherapy. **(A)** Cell viability (clonogenic survival) of HT-29 cells after exposure to radiotherapy of 2, 4, 6, or 8 Gy. *N* = 4 error bars represent SD. **(B)** Clonogenic survival assay of HT-29 cells. Cells were exposed to 100 or 250 nM seeMet 12 and/or radiotherapy of 2, 4, and 6 Gy, *N* = 4, error bars represent SD. **(C)** Representative pictures of the colonies in the clonogenic survival assay after 14 days for control, monotherapy of seeMet 12 and 0, 2, 4, and 6 Gy radiation and the combination of both treatments. **(D,F,H)** Multicellular HT-29 tumor spheroid assays with 100 or 250 nM seeMet 12 and/or radiotherapy of 2, 4, or 6 Gy. *n* = 10 for control and radiotherapy alone, *n* = 5 for treatment groups, Error bars represent SD. **(E,G,I)** Comparison of the spheroid sizes in panels **(D,F,H)** on day 14. Error bars represent SD. **(J)** Representative images of the spheroids after the different treatments. Size reference bar = 4 μm. Considered to be statistically significant *p < 0.05, **p < 0.01, ***p < 0.001, ****p < 0.0001.

The combination effect between seeMet 12 and external beam radiotherapy was first assessed *in vitro*. The effects of radiotherapy alone was first assessed for 0, 2, 4, 6, and 8 Gy in clonogenic survival assays, demonstrating dose dependent therapeutic effects (Figure 4A). Combination effects of 100 or 250 nM seeMet 12 with various doses of radiotherapy were then assessed in clonogenic survival assays (Figures 4B,C), demonstrating significant potentiating effects of seeMet 12 at 2 and 4 Gy. For example, survival fractions of cells irradiated with 2 Gy in combination with 100 or 250 nM seeMet 12 were reduced by 60 and 79% from cells treated with 2 Gy of radiotherapy alone. Synergy calculations demonstrated synergistic effects at 2 Gy, and additive effects at 4 and 6 Gy. For 2 Gy, CI values were 0.60 and 0.46 for 100 and 250 nM seeMet 12, respectively. For 4 Gy, CI values were 0.91 and 0.97, and for 6 Gy CI values were 0.86 and 0.91 for 100 and 250 nM seeMet 12 respectively.

Growth inhibitory effects in 3D multicellular tumor spheroids were then assessed for combinations of radiotherapy (0, 2, 4, or 6 Gy) and seeMet 12 (250 nM) (Figures 4D–J). Results were in line with the clonogenic survival assays, with 4 Gy radiotherapy in combination with 250 nM seeMet 12 demonstrating the strongest potentiating effects. Here, 250 nM seeMet 12 in combination with 4 Gy resulted in a reduction of spheroid sizes of 26 and 22% from cells treated with radiotherapy or seeMet 12 alone, respectively.

### SeeMet 12 Potentiates Radiotherapy in Colorectal Cancer Xenografts *in vivo*

**FIGURE 5:**
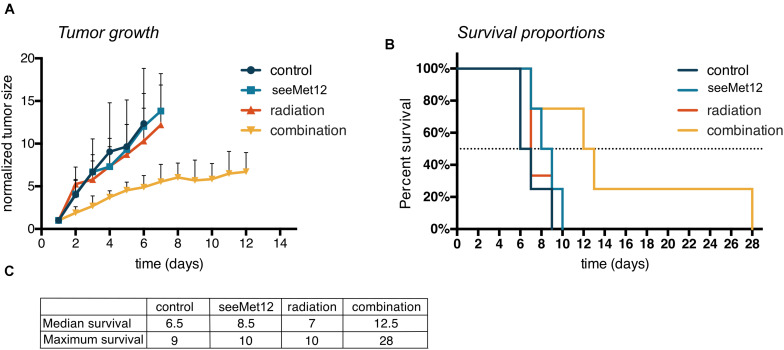
Characterization of seeMet 12 therapy in combination with external radiotherapy in colorectal cancer xenografts. **(A)** HT-29 tumor growth followed over time, until the first animal in each group was sacrificed. HT-29 xenografted mice received treatment with 5× seeMet 12 and/or radiotherapy of 3 × 2Gy. Control animals received placebo treatment. *n* = 4 per group (control, seeMet 12 and combination treatment), *n* = 3 per group (radiation treatment), Error bars represent SD. **(B)** Survival proportions of the different treatment groups, *n* = 3–4 per group. **(C)** Survival 50% and maximum survival for the different treatment regiments of the mouse xenograft study.

Results from the *in vivo* proof-of-concept study in HT-29 xenograft bearing mice can be seen in Figure 5 and Supplementary Figures 1A–D. A combination of seeMet 12 and radiotherapy increased the median survival by 79% compared to radiotherapy alone, and tripled maximum survival. Median survival for control animals was 6.5 days, for seeMet-treated animals 8.5 days, for radiotherapy treated animals 7 days, and for combination treated animals 12.5 days. Consequently, combination treatment increased median survival with 92% from control and 79% from radiotherapy. Maximum survival for control animals was 9 days, for seeMet-treated animals 10 days, for radiotherapy treated animals 10 days, and for combination treated animals 28 days (Figures 5B,C). Consequently, combination treatment tripled maximum survival compared to all other groups. Administration of drug and/or radiotherapy did not influence animal weight (Supplementary Figure 1D), and no adverse effects from treatments were observed.

In addition, molecular effects of seeMet 12 treatment and/or radiotherapy were also assessed *ex vivo* through histological and immunohistochemical analyses of tumor xenografts (Supplementary Figures 1E,F). All tumors displayed high and homogenous cMet stainings, assessed as +++ in staining intensities. No differences between the groups were observed for the area of necrosis or Ki67 proliferation index. However, increased apoptosis was observed in the combination treated xenografts compared to monotherapies, albeit with large variances within the groups.

## Discussion

The tyrosine kinase receptor cMet is an interesting target for cancer therapy, since it holds pivotal roles in proliferation, tumor invasiveness, and drug resistance (1, 28). With these involvements of cMet in cancer progression and therapy, combination strategies using cMet inhibitors may bring beneficial effects for existing cancer therapies, not only by adding the effects of the monotherapy but also by preventing or reducing treatment resistance or inducing synergistic combination effects. Consequently, in this study, we evaluated the effects of the anti-cMet antibody seeMet 12 in colorectal adenocarcinoma models, as well as its effects in combination with the multi-kinase inhibitor sorafenib or radiotherapy. These treatment combinations are clinically relevant to study, as sorafenib is currently in clinical trials for colorectal cancer, and radiotherapy has been a cornerstone for treatment of this patient group for decades (13, 17).

We have previously developed a panel of monoclonal anti-cMet antibodies for potential cancer therapy and diagnosis (7). From these, three promising IgG1 antibodies, all binding to different epitopes of the cMet α-chain, were purified from hybridoma supernatants after the cells adapted well to growth in serum free media, and were used for characterizations in the present study. The antibodies (seeMet 12, seeMet 13 and seeMet 18) were first were assessed for antigen specificity and effects on cell viability in the two cMet positive gastrointestinal cancer cell lines HT-29 and MKN-45 (Figure 1). Specificity assays demonstrated that radiolabeled antibody binding could be significantly blocked by an excess of the corresponding unlabeled antibody for seeMet 12 and seeMet 18 on both cell lines, whereas blocking of seeMet 13 was not significant in HT-29 cells at these settings. It is however, possible that a larger excess of unlabeled antibodies would have induced a more effective block for this antibody. Moreover, as seeMet 13 has previously been shown to be internalized upon binding (7), this may have influenced the blocking efficacy. Besides demonstrating specific binding of the antibodies, the specificity assay also validated the positive cMet expression on both cell lines, in line with previous studies (29, 30).

In XTT viability assays on both HT-29 and MKN-45 cells, dose-dependent effects for all three antibodies were demonstrated, with a trend of seeMet 12 inducing the greatest effects, followed by seeMet 18. Interestingly, in a previous characterization, the effects of 10 μg/ml seeMet 13 and seeMet 18 on gastric carcinoma SNU-5 cell growth were assessed in cell-count and CellTracker Green assays, demonstrating that seeMet 13 induced a larger inhibition of cell division than seeMet 18 (7). This difference may reflect the different parameters and time-spans assessed in the assays, as well as the different interactions with the specific cell lines.

Taken together, these data confirm the concept of the seeMet antibodies as a therapeutic strategy toward cMet expressing gastrointestinal cancers. This is in line with our previous characterizations, where the effects of several seeMet antibodies were validated in gastric carcinoma as well as e.g., glioma and breast cancer cell lines (7). Moreover, the clear and reproducible data for seeMet 12, with distinct antigen-specific binding and dose dependent therapeutic effects in both cell lines, favored it as the candidate with best performance to be selected for further characterizations in both long- and short-term *in vitro* assays in the present study (Figure 2).

Characterizations of the molecular effects of the seeMet 12 monotherapy demonstrated a slight downregulation of the receptor kinase Met and its phosphorylated form, as well as downstream targets such as the cell signal proteins AKT, ERK, BAD, pSTAT3 and mTor, with more pronounced effects at higher concentrations (Figure 2E). This supports the cMet specific effects obtained by seeMet 12 treatments. The decrease of pSTAT3 with increasing seeMet 12 concentrations indicates increased levels of apoptosis (31), further supported by the observed increase of the apoptosis marker cleaved caspase 3/7 by flow cytometry (Figure 2F), as well as dose-dependent effects on cell migration/proliferation and growth (Figure 2D). This is in line with previous studies, demonstrating that cMet inhibition can result in reduced cell migration and proliferation (32).

Therapeutic effects of the monotherapy were also assessed in a three-dimensional tumor spheroid model (Figure 2C). This model reflects effects of both cell death and growth inhibition in a more *in vivo*-like environment, mimicking the situation of small non-vascularized metastases (33–35). Results were in line with the monolayer assays, demonstrating therapeutic effects of the antibody seeMet 12 in the cMet-positive *in vitro* cancer model. This indicates a therapeutic potential of seeMet 12 as monotherapy in cMet-expressing cancers.

The combination of seeMet 12 and the BRAF inhibitor sorafenib was then assessed as a potential combination strategy, as cMet is often highly overexpressed in colorectal cancers, whereas BRAF mutations are present in approximately 10% of metastatic cases (36). Sorafenib targets both wild type and mutant BRAF, however, the efficacy of sorafenib has been limited by the development of drug resistance. The complete resistance mechanism underlying this is still elusive, though EMT and MET, together with the critical growth factors and signaling pathway involved in the two transition processes, have been shown to play a pivotal role by promoting invasive growth program and protecting cells from apoptosis (11). Consequently, various combination therapies have been explored, using both horizontal and vertical blockades, such as gedatolisib, everolimus, and refametinib. Results have been promising, but the efficacy of sorafenib combined with other molecular targeted drugs still needs further exploration.

In the present study, we hypothesized that seeMet 12 may potentiate sorafenib therapy. The efficacy of sorafenib alone was first assessed in HT-29 (BRAF^V600E^ mutant) cells, demonstrating dose dependent effects on viability (Figure 3A). Combination studies in clonogenic survival assays demonstrated that a concentration of 2 μg/ml sorafenib and seeMet 12 mediated synergistic growth suppression (Figures 3B,C). Moreover, further studies in tumor spheroids demonstrated that the combination with seeMet 12 mediated significant spheroid size reductions compared to sorafenib alone (Figures 3D,E). These results are in line with a recent study in melanoma, where it was demonstrated that HGF/MET signaling contributed to resistance to the BRAF inhibitor vemurafenib via activation of ERK-MAPK and PI3K-AKT, and that pharmacologic inhibition of the cMet/AKT pathway restored the sensitivity (37). Consequently, dual targeting of cMet and BRAF in colorectal cancer may be a promising concept, and should be further explored, in particular since it may also offer hope to overcome resistance of several targeted drugs.

Next, we assessed a combination treatment using seeMet 12 and external beam radiation therapy in colorectal cancer models, as radiotherapy is established as a mainstay of treatment alongside surgery in this cancer type (38). Studies have suggested that cMet may mediate radiation resistance and induce DNA repair through EMT induction and increased PI3K and AKT signaling (10), and that blocking cMet activation might increase the sensitivity of cancer cells to radiation. Consequently, in the present study we hypothesized that seeMet 12 may potentiate effects of radiotherapy. Clonogenic survival assays as well as tumor spheroid models were used in order to assess potential growth inhibitory effects *in vitro* of the combination. Both methods allow extended incubation time (approximately 2 weeks) allowing for detection of late effects caused by ionizing radiation. Results demonstrated that the combination of seeMet 12 and radiotherapy significantly suppressed both the colony forming ability and the growth of HT-29 spheroids (Figure 4). From this we conclude that seeMet 12 possess the potential of augmenting radiotherapy in these settings. This is in line a recent study, where the cMet inhibitor PHA-665752 demonstrated radiosensitizing effects by reducing the cells’ ability to perform homologous recombination, an essential repair pathway of radiation-induced DNA double-strand breaks, by blocking the formation of the RAD51−BRCA2 complex (19). We consider it likely that radiosensitization by seeMet 12 in HT-29 is caused by similar mechanisms, and this should be assessed in more detail in future studies as it was not in the scope of the present study.

In our combination studies with both sorafenib and radiotherapy, combination effects were more pronounced in the 2D clonogenic assays than in the tumor spheroid assays, reflecting the different thresholds, parameters, and time-frames of the assays. 3D cell culture has been reported to be more resistant to radiation and drug therapy than the cells growing as a monolayer (39), attributed to factors such as drug penetration, hypoxia, cell-cell interactions and levels of hererocromatin (40, 41). Thus, it is important to assess treatment efficacy in several models in order to thoroughly evaluate the potential effects of treatments.

Radiotherapy is a long-established treatment and a cornerstone in multimodality treatment of several gastrointestinal tumors, including colorectal cancer. While sorafenib has shown promise, it is so far only approved for the treatment of advanced renal cell carcinoma, hepatocellular carcinoma, AML and advanced thyroid carcinoma. Thus, in the present study we chose to focus on the combination of seeMet 12 with fractionated radiotherapy in a proof-of-concept *in vivo* study in colorectal cancer xenografts.

Interestingly, while both radiotherapy and seeMet 12 demonstrated only minor effects as monotherapies *in vivo*, the combination therapy resulted in 79% increased median survival compared to radiotherapy alone, and tripled maximum survival from 10 to 28 days. These results should be viewed with caution however, as this was a proof-of-concept study with a small number of animals, but are nevertheless encouraging and indicate a promising direction for further research. The *in vivo* results were in agreement with the *in vitro* results, although more pronounced. This was to be expected, since only one treatment dose was given in the *in vitro* studies, whereas the *in vivo* study was designed to obtain maximum effect of the combination, using a setup of 3^∗^2 Gy fractionations of radiotherapy together with 5 separate 100 μg seeMet 12 doses.

IHC analyses of the treated tumors demonstrated strong and unaltered cMet stainings in all groups. This is encouraging, since a potential radiation-induced downregulation of cMet receptors would contradict a combination of radiotherapy and cMet-targeted treatment. Histological and IHC examinations did not reveal any significant differences between the treatment groups for necrosis, mitosis, and cell viability, whereas the combination group indicated increased apoptosis compared to monotherapies (Supplementary Figures 1E,F). Interestingly, one tumor in the combination group demonstrated particularly elevated levels of both necrosis and apoptosis. This was consistent with the dramatic therapeutic effect on tumor size observed in this subject, demonstrating the most reduced tumor growth and longest mouse survival. However, in order to draw any wider conclusions of the molecular effects of the treatments *ex vivo*, longitudinal analyses during and after treatment should be performed in a larger study.

Importantly, administration of drug and/or radiotherapy did not have an effect on the animal weight (Supplementary Figure 1D), and no other adverse effects from treatments were observed. This is in line with recent studies on other bivalent anti-cMET antibodies such as Emibetuzumab and Telisotuzumab. In a recent Phase I dose escalation study of Telisotuzumab, no dose-limiting toxicities were observed, with the most common treatment-related adverse events including hypoalbuminemia and fatigue (42). In a Phase 2 Study of the MET Antibody Emibetuzumab in Combination with Erlotinib, Emibetuzumab plus erlotinib was shown to be well tolerated, with peripheral edema and mucositis as the only adverse events occurring 10% or more frequently relative to erlotinib (43). These initial results are encouraging and indicate that the addition of cMet-binding antibodies such as seeMet 12 may aid in increasing curative rates for radiotherapy.

Combination therapy, in a synergistic or additive manner, has the potential to enhance the efficacy and reduce the resistance of treatment by targeting several mechanisms in cancer while dispersing off-target effects. Furthermore, the use of combination treatments may also reduce the side effects of drug therapies by enabling lower drug doses without compromising the therapeutic efficacy (44). In addition, drug combinations may lead to better outcomes by overcoming patient to patient variability (45). In the present study, we conclude that there is strong evidence that cMet-targeted therapies and their combination with other cancer therapies could be beneficial to improve cancer treatment effectiveness. Furthermore, seeMet 12 is a well-performing lead molecule for developing a novel cMet-targeting drug. However, comprehensive investigations in additional models regarding antibody dosing, fractionations and timing with targeted- or radiation treatment are warranted, as well as detailed analyses of mechanisms of action and potential toxicities in normal tissues in order to proceed with this lead. If further developed, we believe that combination treatment using cMet-targeted therapy has the potential to increase cure rates, reduce treatment toxicity and thereby be of clinical benefit.

## Data Availability Statement

The datasets generated for this study are available on request to the corresponding author.

## Ethics Statement

The animal study was reviewed and approved by Uppsala Animal Testing Ethics Committee.

## Author Contributions

DS, DL, and MN designed the study. DS and MN drafted the manuscript. DS, AM, BV, KP, PM, and JW contributed to experimental studies. DS, AM, KP, JW, PM, BV, DL, and MN contributed to study design, data analysis, and interpretation, and revised the manuscript. All authors contributed to the article and approved the submitted version.

## Conflict of Interest

BV is partially associated with Moravian Biotechnology, the company that participated on development of seeMet monoclonal antibodies. However, the company did not provide financial support for the studies and had no influence on the design, execution, or analysis of the experiments. The remaining authors declare that the research was conducted in the absence of any commercial or financial relationships that could be construed as a potential conflict of interest.

## Supplementary Material

FIGURE S1 HT-29 tumor analysis [*in vivo* **(A–D)** and *ex vivo* **(E,F)**]. **(A)** Individual tumor size at start of the *in vivo* study. **(B)** Tumor size over time for each tumor. **(C)** Formalin fixated tumors, dissected at experimental endpoint (when reaching a tumor size of 1,000 mm^3^). **(D)** Animal weight during the *in vivo* study. *n* = 4 per group (control, seeMet 12 and combination treatment), *n* = 3 per group (radiation treatment). **(E)** *Ex vivo* immunohistochemistry analysis for Ki67, necrosis, mitosis and apoptosis (Annexin V. expression). Error bars represent SD. **(F)** Representative tumor images of hematoxylin and eosin stainings and immunohistochemical staining for (H&E), cMet, Ki67 and Aneexin. V. Size reference bar = 20 μm.

## References

[1] Sierra JR Tsao MS. c-MET as a potential therapeutic target and biomarker in cancer. *Ther Adv Med Oncol.* (2011) 3(1 Suppl):S21–35. 22128285 10.1177/1758834011422557 PMC3225018

[2] Lengyel E Prechtel D Resau JH Gauger K Welk A Lindemann K c-MET overexpression in node-positive breast cancer identifies patients with poor clinical outcome independent of Her2/neu. *Int J Cancer.* (2005) 113:678–82. 10.1002/ijc.20598 15455388

[3] Olivero M Rizzo M Madeddu R Casadio C Pennacchietti S Nicotra MR Overexpression and activation of hepatocyte growth factor/scatter factor in human non-small-cell lung carcinomas. *Br J Cancer.* (1996) 74:1862–8. 10.1038/bjc.1996.646 8980383 PMC2074802

[4] Nakajima M Sawada H Yamada Y Watanabe A Tatsumi M Yamashita J The prognostic significance of amplification and overexpression of c-met and c-erb B-2 in human gastric carcinomas. *Cancer.* (1999) 85:1894–902. 10.1002/(sici)1097-0142(19990501)85:9<1894::aid-cncr3>3.0.co;2-j 10223227

[5] Chen X Guan Z Lu J Wang H Zuo Z Ye F Synergistic antitumor effects of cMet inhibitor in combination with anti-VEGF in colorectal cancer patient-derived xenograft models. *J Cancer.* (2018) 9:1207–17. 10.7150/jca.20964 29675102 PMC5907669

[6] Safaie Qamsari E Safaei Ghaderi S Zarei B Dorostkar R Bagheri S Jadidi-Niaragh F The c-Met receptor: implication for targeted therapies in colorectal cancer. *Tumour Biol.* (2017) 39:1010428317699118. 10.1177/1010428317699118 28459362

[7] Wong JS Warbrick E Vojtesk B Hill J Lane DP. Anti-c-Met antibodies recognising a temperature sensitive epitope, inhibit cell growth. *Oncotarget.* (2013) 4:1019–36. 10.18632/oncotarget.1075 23859937 PMC3759663

[8] Garajova I Giovannetti E Biasco G Peters GJ. c-Met as a target for personalized therapy. *Transl Oncogenomics.* (2015) 7 (Suppl. 1):13–31. 10.4137/tog.s30534 26628860 PMC4659440

[9] Delitto D Vertes-George E Hughes SJ Behrns KE Trevino JG. c-Met signaling in the development of tumorigenesis and chemoresistance: potential applications in pancreatic cancer. *World J Gastroenterol.* (2014) 20:8458–70. 10.3748/wjg.v20.i26.8458 25024602 PMC4093697

[10] Bhardwaj V Cascone T Cortez MA Amini A Evans J Komaki RU Modulation of c-Met signaling and cellular sensitivity to radiation: potential implications for therapy. *Cancer.* (2013) 119:1768–75. 10.1002/cncr.27965 23423860 PMC3648606

[11] Chen J Jin R Zhao J Liu J Ying H Yan H Potential molecular, cellular and microenvironmental mechanism of sorafenib resistance in hepatocellular carcinoma. *Cancer Lett.* (2015) 367:1–11. 10.1016/j.canlet.2015.06.019 26170167

[12] Dong N Shi X Wang S Gao Y Kuang Z Xie Q M2 macrophages mediate sorafenib resistance by secreting HGF in a feed-forward manner in hepatocellular carcinoma. *Br J Cancer.* (2019) 121:22–33. 10.1038/s41416-019-0482-x 31130723 PMC6738111

[13] Samalin E Bouche O Thezenas S Francois E Adenis A Bennouna J Sorafenib and irinotecan (NEXIRI) as second- or later-line treatment for patients with metastatic colorectal cancer and KRAS-mutated tumours: a multicentre Phase I/II trial. *Br J Cancer.* (2014) 110:1148–54. 10.1038/bjc.2013.813 24407191 PMC3950852

[14] Wilhelm S Carter C Lynch M Lowinger T Dumas J Smith RA Discovery and development of sorafenib: a multikinase inhibitor for treating cancer. *Nat Rev Drug Discov.* (2006) 5:835–44. 10.1038/nrd2130 17016424

[15] Llovet JM Villanueva A Lachenmayer A Finn RS. Advances in targeted therapies for hepatocellular carcinoma in the genomic era. *Nat Rev Clin Oncol.* (2015) 12:436. 10.1038/nrclinonc.2015.121 26099984

[16] Berasain C. Hepatocellular carcinoma and sorafenib: too many resistance mechanisms? *Gut.* (2013) 62:1674–5. 10.1136/gutjnl-2013-304564 23481262

[17] Thompson MK Poortmans P Chalmers AJ Faivre-Finn C Hall E Huddart RA Practice-changing radiation therapy trials for the treatment of cancer: where are we 150 years after the birth of Marie Curie? *Br J Cancer.* (2018) 119:389–407. 10.1038/s41416-018-0201-z 30061587 PMC6117262

[18] Hu SY Duan HF Li QF Yang YF Chen JL Wang LS Hepatocyte growth factor protects endothelial cells against gamma ray irradiation-induced damage. *Acta Pharmacol Sin.* (2009) 30:1415–20. 10.1038/aps.2009.133 19749787 PMC4007330

[19] Medova M Aebersold DM Zimmer Y. MET inhibition in tumor cells by PHA665752 impairs homologous recombination repair of DNA double strand breaks. *Int J Cancer.* (2012) 130:728–34. 10.1002/ijc.26058 21400509

[20] Fan S Wang JA Yuan RQ Rockwell S Andres J Zlatapolskiy A Scatter factor protects epithelial and carcinoma cells against apoptosis induced by DNA-damaging agents. *Oncogene.* (1998) 17:131–41. 10.1038/sj.onc.1201943 9674697

[21] Zhao Y Liu P Zhang N Chen J Landegger LD Wu L Targeting the cMET pathway augments radiation response without adverse effect on hearing in NF2 schwannoma models. *Proc Natl Acad Sci USA.* (2018) 115:E2077–84. 29440379 10.1073/pnas.1719966115 PMC5834719

[22] Jin H Yang R Zheng Z Romero M Ross J Bou-Reslan H MetMAb, the one-armed 5D5 anti-c-Met antibody, inhibits orthotopic pancreatic tumor growth and improves survival. *Cancer Res.* (2008) 68:4360–8. 10.1158/0008-5472.can-07-5960 18519697

[23] Pacchiana G Chiriaco C Stella MC Petronzelli F De Santis R Galluzzo M Monovalency unleashes the full therapeutic potential of the DN-30 anti-Met antibody. *J Biol Chem.* (2010) 285:36149–57. 10.1074/jbc.m110.134031 20833723 PMC2975237

[24] Greenall SA Gherardi E Liu Z Donoghue JF Vitali AA Li Q Non-agonistic bivalent antibodies that promote c-MET degradation and inhibit tumor growth and others specific for tumor related c-MET. *PLoS One.* (2012) 7:e34658. 10.1371/journal.pone.0034658 22511956 PMC3325269

[25] Harlow E Lane D. *Antibodies: A Laboratory Manual.* Cold Spring Harbor: CSHL Press (1988).

[26] Liang CC Park AY Guan JL. In vitro scratch assay: a convenient and inexpensive method for analysis of cell migration in vitro. *Nat Protoc.* (2007) 2:329–33. 10.1038/nprot.2007.30 17406593

[27] Yao HP Feng L Weng TH Hu CY Suthe SR Mostofa AGM Preclinical efficacy of anti-RON antibody-drug conjugate Zt/g4-MMAE for targeted therapy of pancreatic cancer overexpressing RON receptor tyrosine kinase. *Mol Pharm.* (2018) 15:3260–71. 10.1021/acs.molpharmaceut.8b00298 29944378

[28] Firtina Karagonlar Z Koc D Iscan E Erdal E Atabey N. Elevated hepatocyte growth factor expression as an autocrine c-Met activation mechanism in acquired resistance to sorafenib in hepatocellular carcinoma cells. *Cancer Sci.* (2016) 107:407–16. 10.1111/cas.12891 26790028 PMC4832867

[29] Ma JA Hu C Li W Ren J Zou F Zhou D Downregulation of c-Met expression does not enhance the sensitivity of gastric cancer cell line MKN-45 to gefitinib. *Mol Med Rep.* (2015) 11:2269–75. 10.3892/mmr.2014.2948 25395073

[30] Qiu P Wang S Liu M Ma H Zeng X Zhang M Norcantharidin inhibits cell growth by suppressing the expression and phosphorylation of both EGFR and c-Met in human colon cancer cells. *BMC Cancer.* (2017) 17:55. 10.1186/s12885-016-3039-x 28086832 PMC5237309

[31] Al Zaid Siddiquee K Turkson J. STAT3 as a target for inducing apoptosis in solid and hematological tumors. *Cell Res.* (2008) 18:254–67. 10.1038/cr.2008.18 18227858 PMC2610254

[32] Gonzalez A Broussas M Beau-Larvor C Haeuw JF Boute N Robert A A novel antagonist anti-cMet antibody with antitumor activities targeting both ligand-dependent and ligand-independent c-Met receptors. *Int J Cancer.* (2016) 139:1851–63. 10.1002/ijc.30174 27144973

[33] Yamada KM Cukierman E. Modeling tissue morphogenesis and cancer in 3D. *Cell.* (2007) 130:601–10. 10.1016/j.cell.2007.08.006 17719539

[34] Kimlin LC Casagrande G Virador VM. In vitro three-dimensional (3D) models in cancer research: an update. *Mol Carcinog.* (2013) 52:167–82. 10.1002/mc.21844 22162252

[35] Hirschhaeuser F Menne H Dittfeld C West J Mueller-Klieser W Kunz-Schughart LA. Multicellular tumor spheroids: an underestimated tool is catching up again. *J Biotechnol.* (2010) 148:3–15. 10.1016/j.jbiotec.2010.01.012 20097238

[36] Barras D. *BRAF* mutation in colorectal cancer: an update. *Biomark Cancer.* (2015) 7 (Suppl. 1):9–12. 26396549 10.4137/BIC.S25248 PMC4562608

[37] Zubovych IO Sethi A Kulkarni A Tagal V Roth MG. A novel inhibitor of topoisomerase I is selectively toxic for a subset of non-small cell lung cancer cell lines. *Mol Cancer Ther.* (2016) 15:23–36. 10.1158/1535-7163.mct-15-0458 26668189 PMC4707128

[38] Hafner MF Debus J. Radiotherapy for colorectal cancer: current standards and future perspectives. *Visc Med.* (2016) 32:172–7. 10.1159/000446486 27493944 PMC4945782

[39] Dickreuter E Cordes N. The cancer cell adhesion resistome: mechanisms, targeting and translational approaches. *Biol Chem.* (2017) 398:721–35. 10.1515/hsz-2016-0326 28002024

[40] Kapalczynska M Kolenda T Przybyla W Zajaczkowska M Teresiak A Filas V 2D and 3D cell cultures–a comparison of different types of cancer cell cultures. *Arch Med Sci.* (2018) 14:910–9. 30002710 10.5114/aoms.2016.63743 PMC6040128

[41] Storch K Eke I Borgmann K Krause M Richter C Becker K Three-dimensional cell growth confers radioresistance by chromatin density modification. *Cancer Res.* (2010) 70:3925–34. 10.1158/0008-5472.can-09-3848 20442295

[42] Strickler JH LoRusso P Salgia R Kang Y-K Yen CJ Lin C-C Phase I dose-escalation and -expansion study of telisotuzumab (ABT-700), an anti–c-Met antibody, in patients with advanced solid tumors. *Mol Cancer Ther.* (2020) 19:1210. 10.1158/1535-7163.mct-19-0529 32127466

[43] Scagliotti G Moro-Sibilot D Kollmeier J Favaretto A Cho EK Grosch H A randomized-controlled phase 2 study of the MET antibody emibetuzumab in combination with erlotinib as first-line treatment for EGFR mutation-positive NSCLC patients. *J Thorac Oncol.* (2020) 15:80–90. 10.1016/j.jtho.2019.10.003 31622732

[44] Bayat Mokhtari R Homayouni TS Baluch N Morgatskaya E Kumar S Das B Combination therapy in combating cancer. *Oncotarget.* (2017) 8:38022–43. 10.18632/oncotarget.16723 28410237 PMC5514969

[45] Palmer AC Sorger PK. Combination cancer therapy can confer benefit via patient-to-patient variability without drug additivity or synergy. *Cell.* (2017) 171:1678–91.e13. 29245013 10.1016/j.cell.2017.11.009 PMC5741091

