# Supplementary figures and images for: The Novel Anti-cMet Antibody seeMet 12 Potentiates Sorafenib Therapy and Radiotherapy in a Colorectal Cancer Model
Source: Front Oncol. 2020 Sep 11;10:1717. doi: 10.3389/fonc.2020.01717 (PMC7516085; doi:10.3389/fonc.2020.01717)

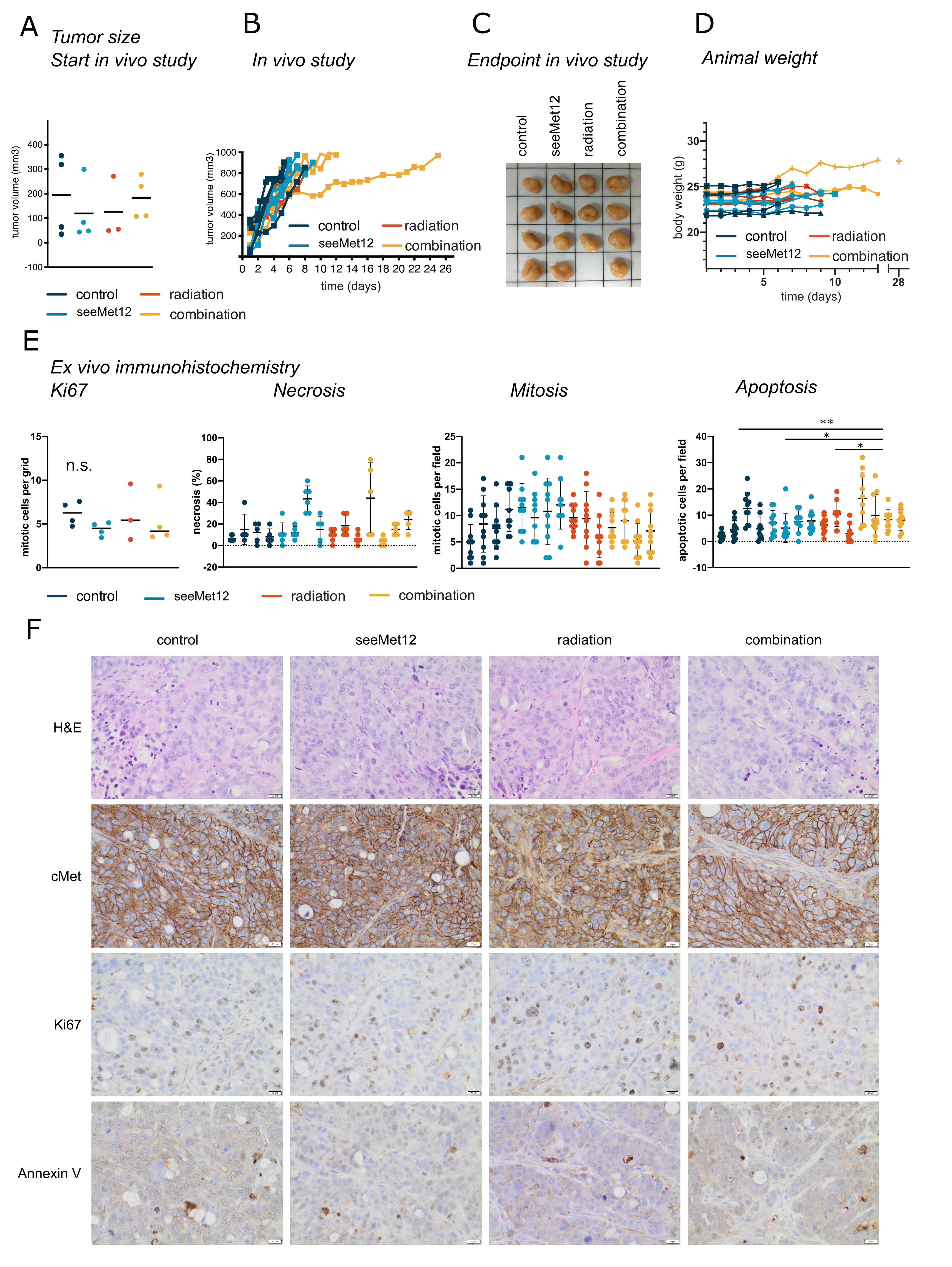

Supplement: FIGURE S1 — HT-29 tumor analysis [in vivo (A–D) and ex vivo (E,F)]. (A) Individual tumor size at start of the in vivo study. (B) Tumor size over time for each tumor. (C) Formalin fixated tumors, dissected at experimental endpoint (when reaching a tumor size of 1,000 mm3). (D) Animal weight during the in vivo study. n = 4 per group (control, seeMet 12 and combination treatment), n = 3 per group (radiation treatment). (E) Ex vivo immunohistochemistry analysis for Ki67, necrosis, mitosis and apoptosis (Annexin V. expression). Error bars represent SD. (F) Representative tumor images of hematoxylin and eosin stainings and immunohistochemical staining for (H&E), cMet, Ki67 and Aneexin. V. Size reference bar = 20 μm. [file Image_1.PNG]
